# Identifying structural barriers to an effective HIV response: using the National Composite Policy Index data to evaluate the human rights, legal and policy environment

**DOI:** 10.7448/IAS.16.1.18000

**Published:** 2013-04-26

**Authors:** Sofia Gruskin, Laura Ferguson, Tobias Alfven, Deborah Rugg, Greet Peersman

**Affiliations:** 1 Program on Global Health and Human Rights, Institute for Global Health, University of Southern California, Los Angeles, CA, USA; 2 UNAIDS, Geneva, Switzerland; 3 Payson Center for International Development and Technology Transfer, Law School, Tulane University, New Orleans, LA, USA

**Keywords:** AIDS, HIV, human rights, key populations, law, policy, structural barriers

## Abstract

**Introduction:**

Attention to the negative effects of structural barriers on HIV efforts is increasing. Reviewing national legal and policy environments with attention to the international human rights commitments of states is a means of assessing and providing focus for addressing these barriers to effective HIV responses.

**Methods:**

Law and policy data from the 171 countries reporting under the Declaration of Commitment from the 2001 United Nations General Assembly Special Session on HIV/AIDS were analyzed to assess attention to human rights in national legal and policy environments as relevant to the health and rights of key populations such as people who inject drugs, men who have sex with men and sex workers.

**Results:**

Seventy-eight governments and civil society in 106 countries report the existence of laws and policies which present obstacles to accessing HIV services for key populations. Laws and policies which positively affect access to HIV-related services, in and of themselves constituting structural interventions, were also reported. The dissonance between laws and how this impacts the availability and use of HIV-related services deserve greater attention.

**Conclusions:**

Recognition of the harms inherent in laws that constitute structural barriers to effective HIV responses and the potential positive role that a supportive legal environment can play suggests the need for legal reform to ensure an enabling regulatory framework within which HIV services can be effectively delivered and used by the populations who need them. Moving beyond laws and policies, further efforts are required to determine how to capture information on the range of structural barriers. Teasing apart the impact of different barriers, as well as the structural interventions put in place to address them, remains complicated. Capturing the impact of policy and legal interventions can ultimately support governments and civil society to ensure the human rights of key populations are protected in national HIV responses.

## Introduction

There is increasing attention to “structural barriers” in the HIV literature and to the ways these inhibit the effectiveness of HIV responses at national and local levels. Despite the proliferation of rhetoric on “structural barriers”, a common definition does not yet exist. Specificity regarding which structural barriers are an issue is key to determining which interventions are needed to address them, and to evaluating their effects. Descriptions of structural barriers include a variety of factors, but commentators generally agree that laws and policies are key parts of the environment impacting both positively and negatively on national HIV responses [1,2].

Reviewing national legal and policy environments with attention to the international human rights commitments of states is a means of assessing and providing focus for addressing legal and policy barriers to effective HIV responses. International human rights law constitutes countries’ over-arching obligations and therefore provides a legal framework within which national laws, policies and programmes can not only be formulated but assessed. The reasons to pay attention to human rights in the HIV response are well known [3]. They include the international legal obligation to ensure human rights are promoted and not violated in the context of HIV efforts, the moral obligation to do no harm and, notably, their importance for putting into place an enabling legal and policy environment to support effective HIV programming.

Marginalized populations are known to be disproportionately affected by HIV in many places and particularly susceptible to the adverse impacts of structural barriers [4,5]. Governments put into place a wide range of obstructive laws ranging from regulation or criminalization of specific behaviours to the arrest and detention of those providing services to people thought to be engaged in illegal behaviours. In many jurisdictions, not only is injecting drugs criminalized but if services are on offer providers may be required to report a person's name and other personal information to the police or other government agency if a client is suspected of using illegal drugs. Furthermore, where needle exchange programmes are outlawed, injecting drug users’ access to safe injecting equipment is undermined, potentially increasing the sharing of injecting equipment. Access to HIV-related information and services is known to be impeded among men who have sex with men (MSM) where sodomy statutes exist, particularly where police and other authorities can harass men who have sex with men with impunity. Sex workers have been driven underground and avoided services for fear of losing their livelihood where sex work is criminalized, especially where health services share personal information with the police and other authorities [6–9]. As the impact of impediments to effective HIV prevention efforts are increasingly recognized, attention to improving the legal and policy context within which HIV interventions to support key populations are implemented is gaining traction at global and national levels.

Recognition of the importance of structural barriers for the success or failure of HIV programmes has also drawn attention to the inadequacy of efforts to collect relevant information about how these barriers interfere and how they can best be addressed in systematic and meaningful ways [10]. This is particularly true in the case of policy data.

Below we seek to identify specific laws and policies that are likely to impede or strengthen national HIV responses. Areas where further work is still needed are highlighted. Findings are situated within the broader body of work to address structural barriers to an effective HIV response.

## Structural barriers and interventions relevant to an effective HIV response

In the context of HIV, the most general understanding of “structural barriers” encompasses contextual factors that exacerbate vulnerability to HIV infection or impede access to HIV-related services (i.e. prevention, treatment, care and support) [11]. Various attempts have been made to classify “structural barriers” to facilitate the targeting of interventions to address them; a few of the most prominent are outlined below.

Blankenship *et al*. create a system of classification of structural interventions based on whether the primary aim is to affect the availability, acceptability or accessibility of services; interventions are further classified by their primary target audience: individual, organizational, or environmental [12]. They classify the use of laws and policies as relevant to the accessibility of services, targeted primarily at what they term the environmental level. Sweat and Denison's classification of structural barriers also focus on the level at which they operate starting with superstructural factors (e.g. economic development) that affect national-level structural factors (e.g. laws and policies), which influence environmental factors (e.g. living conditions) that in turn shape the experiences of individuals (e.g. use of health services) [13]. Barnett and Whiteside have suggested a similar continuum of structural barriers [14]. Parker *et al*. propose four categories of structural barriers and facilitators, but only as relevant to HIV prevention: economic (under)development and poverty; mobility; gender inequalities; and the effects of policies on HIV vulnerability and transmission [15].

One element common to all of these models is recognition of the influence that laws and policies exert on national HIV responses. Harmful laws and policies raise a host of human rights concerns and create structural barriers which inhibit an effective HIV response, especially with regard to populations that are already marginalized. In short, the legal and policy environment influences the availability of HIV services as well as the degree to which they are responsive to individual needs [16].

Structural interventions in the field of HIV encompass efforts to effect change in environments within which behaviours occur but do not attempt to change individual-level knowledge, attitudes or patterns of social interaction [17]. They can be designed to address or overcome any recognized structural barrier with the aim of removing impediments to healthy behaviours and improving access to services by individuals. This can encompass a wide range of interventions including stigma reduction, micro-finance programmes to alleviate poverty, provision of safe housing, mobilization of different communities and legal reform [18]. In this article, we focus on the role of laws and policies as examples of structural interventions.

## Methods

The 2001 United Nations General Assembly Special Session (UNGASS) Declaration of Commitment (DOC) on HIV/AIDS emphasized the centrality of human rights for an effective HIV response [4]. Although the commitments made in the DOC are not themselves legally binding, the UNGASS process provides a clear mandate for countries to collect and report quantitative and law and policy data [19]. Countries submit reports to UNAIDS every two years on their progress towards fulfilling the DOC. Using data from 2010 we undertook a descriptive analysis of the reported existence of specified HIV-related laws and policies relevant to people who use drugs, men who have sex with men and sex workers, and reviewed narrative comments relating to their content, implementation and impacts.

The National Composite Policy Index (NCPI), one part of the UNGASS report, contains a range of questions which can provide insight into laws and policies which act as structural factors (whether barriers or facilitators) affecting the HIV response within countries, especially with regard to key populations such as people who use drugs, men who have sex with men and sex workers.^1^ It consists of two parts: one is filled out by government and the other by representatives of non-governmental sources,^2^ in most countries defined as civil society, and therefore in this paper called “civil society”. Some questions appear in both parts so as to elicit responses from both government and civil society, and we compared these responses when relevant.

The NCPI is compiled through desk reviews and consultation with stakeholders ranging from government officials to groups of people living with HIV, each of whom is assumed to be most knowledgeable about the topics covered.^3^ A process is suggested for negotiation between government and civil society about the final responses submitted. The NCPI is ultimately vetted and submitted by the government, and there is understood to be country ownership of the data submitted. The NCPI was the highest reported UNGASS indicator in the last three reporting rounds, with 171 countries reporting in 2010 out of the 182 countries that submitted UNGASS reports.

## Results

### Obstructive laws and policies

Consistent across the responses of governments and civil society, a very high proportion of countries reported the existence of laws/policies that create obstacles to accessing HIV services in 2010.

The reported content of these obstructive laws varies widely. Some of the legal impediments noted are very broad such as those described by Burundi's civil society: “The criminal code punishes homosexuality, drug consumption and prostitution. It provides for fines and terms of imprisonment” [20]. Egyptian civil society also describes “Sex between unmarried couples is legal if it is between consenting adults who are not married to other individuals, and the sexual act takes place in a private location with no monetary exchange. If any of the above conditions are absent authorities may intervene and the sexual act is punishable” [21].

Some countries, including Brunei Darussalam and Sri Lanka, noted that even though application of such obstructive laws and policies is rare, their mere existence can impede efforts to address HIV [22,23].

Civil society respondents are generally more likely than governments to report the existence of laws/policies that create obstacles to accessing HIV services. While civil society actors are certainly more likely to feel the effects of these obstacles, these differences in reporting may also signal a lack of clarity on the part of governments as to what constitutes a legal/policy obstacle to accessing HIV-related services. Alternatively this could illustrate over-enthusiasm by these same governments to report the existence of an appropriate legal and policy framework.

Irrespective of the difference in responses across regions and respondents, the high percentage of countries that recognize the existence of such barriers is of concern.

**Figure 1:**
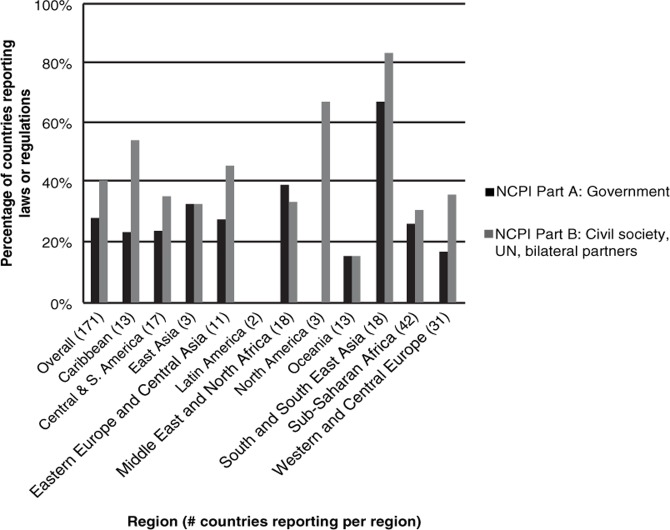
Percentage of countries reporting laws or regulations that create obstacles to accessing HIV services for injecting drug users.

**Figure 2:**
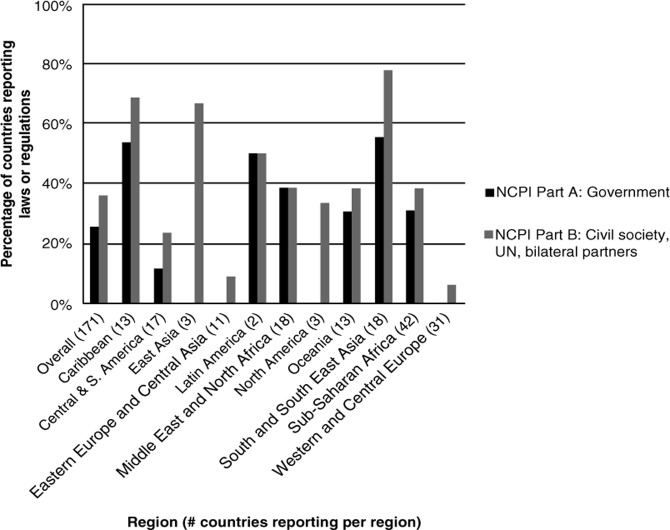
Percentage of countries reporting laws or regulations that create obstacles to accessing HIV services for men who have sex with men.

**Figure 3:**
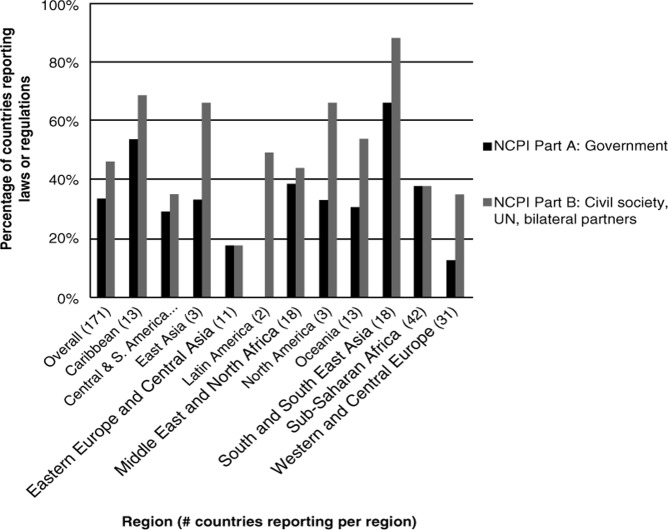
Percentage of countries reporting laws or regulations that create obstacles to accessing HIV services for sex workers.

Figures 1–3 below show reported legal/policy obstacles for injecting drug users, men who have sex with men and sex workers.

Globally, 48 national governments and civil society respondents from 68 countries report the existence of laws or regulations that create obstacles to accessing HIV services for injecting drug users as shown in Figure 1 above. A particularly high proportion of countries in South and South-East Asia report their existence. The lack of reporting of obstacles in Latin America is to be cautiously interpreted as only two countries in this region submitted reports.

The most often-cited obstacle to accessing HIV services for injecting drug users, in low-, middle- and high-income countries alike, is the application of criminal penalties to drug use which acts as an impediment to the distribution of sterile injecting equipment and the provision of HIV-related services. For example, Australian civil society notes that “All states and territories apply criminal penalties to some forms of drug use. Although Australia has been at the forefront of harm reduction strategies for injecting drug use, these criminal penalties sometimes present significant obstacles in the provision of treatment, care and support for people who use illicit drugs” [24].

Figure 2 shows that 44 governments and civil society respondents in 62 countries report the existence of laws or regulations that create obstacles to accessing HIV services for men who have sex with men, with particularly high percentages of countries reporting their existence in the Caribbean and South and South-East Asia.

The obstacles cited as affecting men who have sex with men vary widely and include everything from mandatory HIV testing on conviction of “consensual buggery” (Trinidad & Tobago) to religious conviction that homosexuality is immoral and thus intolerable (Gambia, Egypt) [21,25, 26].

Malaysian civil society describes harsh penalties for insertion of the penis into the anus or the mouth, with the result that “spas and massage centres refuse to supply condoms for fear of legal action being taken on them resulting in the loss of their operating licence and depriving them of business” [27].

While the content of these laws varies, their impacts are similar: they all impede men who have sex with men from accessing the services they require for effective HIV prevention, care and treatment, including access to condoms and sexual health services. This is particularly true where infringement of these laws is punishable by death.

Figure 3 shows that globally, 59 governments and civil society respondents in 80 countries report the existence of legal or regulatory impediments affecting sex workers. A markedly low percentage of countries in Eastern Europe and Central Asia report the existence of such barriers, while South and South-East Asia once again constitutes the region with the highest percentage of countries reporting their existence.

The types of legal and policy barriers to accessing HIV services reported by countries is particularly wide-ranging for sex workers, perhaps reflecting the range of regulatory options in use with regard to sex work. Across diverse settings, many countries described the deleterious impact of direct criminalization of sex work on access to HIV-related services [21,28]. Civil society in the UK noted that proposals to criminalize payment for sex might have additional negative impact on sex workers’ health. Senegal cites a minimum age limit for practising sex work that might impede access to services for younger sex workers while Sri Lanka describes possession of condoms as “proof” of engagement in sex work and Paraguay reports mandatory HIV testing for sex workers [23,29, 30].

The government in Indonesia notes that “the local government bylaws closing prostitution complexes resulted in the spread of street prostitution and make it difficult for local health departments to provide services for sexually transmitted disease control/condom promotion” [31]. These varied examples all constitute ways of impeding sex workers’ access to HIV-related services.

Even when affected populations are prepared to access services, the legal and policy environment may nonetheless constrain the services on offer to them. Bangladesh and the Philippines both highlight that their legal frameworks pose a barrier to the provision of services for people engaged in “illegal behaviours”, for example, police harassment of outreach workers working with key populations or arrest of health workers supplying clean injecting equipment to drug users [32,33]. The government of Saint Kitts and Nevis report that “MSMs feel intimidated due to a perceived discrimination and sex workers go into hiding. No one tries to help as they can be charged and even convicted of aiding and abetting a criminal offence …” [34].

Globally, a remarkable number of countries report the existence of laws/policies which present obstacles to accessing HIV services for key populations. Although there is some regional variation, this constitutes a serious constraint to national HIV responses worldwide.

### “Protective” laws and policies

While laws and policies can be significant structural barriers, regulations can also be used to positive effect, constituting structural interventions to promote access to HIV-related services and to change social norms. These include, for example, non-discrimination laws or policies which specify protections for key populations. A broad range of approaches exist.

Iranian civil society highlights efforts to protect drug users from discrimination: “The law does not consider drug users receiving treatment to be offenders. On harm reduction, a directive has been issued by the chief of the Judiciary whereby the judges are ordered not to obstruct harm reduction interventions” [35]. In considering protections for men who have sex with men, Mexican civil society explained that “In the Federal District the enforcement of a law that protects the right of homosexual men and lesbian women to be married and adopt children has been registered, which favours the environment, as men who have sex with men are the population most affected by HIV and this law is a step forward in the right sense of the fight against homophobia” [36]. In Spain, protection from discrimination for MSM is promoted through homophobia being a designated “aggravant of a felony” [37]. And civil society in Germany outlined the protections conferred on sex workers by laws which “protect sex workers of violence, offer [sic] legal framework of professional sex work including health insurance” [38].

The degree of protection from discrimination that any such laws or policies may confer depends on their specific content, the degree to which they are implemented, and the availability of mechanisms for redress in case of violations.

Many countries reported barriers to the effectiveness of protective laws and policies. Civil society respondents in Oman, for example, noted that “There is general consensus that non-discrimination provisions and regulations do exist, largely within the framework of the National Health Strategy and the National AIDS Strategy, but that the mechanisms for implementation and enforcement are unclear/not-known” [39]. Civil society in El Salvador reported that “In general they [protective laws and policies] exist but have limited applicability. They don't adapt. They are also not disseminated” [40].

### Conflicts in law

The impacts on HIV-related services of conflicting laws deserve greater attention. Overall, 68% of governments and 71% of civil society respondents that reported the existence of “protective” laws or regulations, also report the existence of regulatory obstacles to accessing HIV-related services. The Ukraine government acknowledged that “According to the experts, the laws exist in Ukraine but they are often not supported by subordinate legislation … The situation often arises when the provisions of one law contradict those of another. This leads to a situation when laws exist formally but are not enforced, while representatives of risk groups face discrimination in their attempts to receive health care, education, employment, etc.” [41]. A similar issue is noted in Mexico where some municipal laws on mandatory HIV testing for sex workers are in opposition to federal non-discrimination laws and in Malaysia where carrying syringes and needles outside healthcare settings is illegal despite the existence of a government mandated harm reduction programme, which presumably would include the provision of safe injecting equipment [27,36]. The Indonesian government noted that “The Law on Narcotics does not support harm reduction services such as needle/syringe exchange. Special arrangements and negotiations with the local police are needed to enable needle/syringe exchange services to be provided, impeding the general provision of these services for drug users” [31]. Highlighting such conflicts in national legal and policy environments can help focus legal reform efforts and advocacy to target change.

## Discussion

The NCPI data draw attention to the widespread existence of laws and policies that constitute structural barriers to effective HIV responses for key populations around the world. For people who use drugs, the barriers are fairly similar across different national settings with most reported barriers constituting impediments to accessing harm reduction services. For men who have sex with men a diverse range of obstacles exist across different countries including mandatory HIV testing and barriers to accessing condoms. For sex workers, wide-ranging regulatory barriers were cited including restrictions on who can engage in sex work and where sex work can be carried out. The NCPI also highlights “protective” laws and policies designed to promote an effective HIV response for key populations, as well as conflicts which exist in policy frameworks.

UNAIDS has underscored the importance of legal and policy environments which respect human rights to ensure an effective HIV response. The UNAIDS five-year strategy includes “advancing human rights and gender equality” as one of the three pillars necessary for stopping the HIV epidemic (alongside the pillars of prevention and treatment) [42].

The 2010 report of the UN Special Rapporteur on the Right to Health focuses on criminalization of same-sex relationships, sex work and HIV transmission. Framing his arguments within the obligations under the right to the highest attainable standard of health, he notes that “criminalization impacts detrimentally on health outcomes for individuals, even if the laws around these practices are not enforced, or enforced infrequently” [43]. He recommends decriminalization and the introduction of appropriate monitoring and accountability mechanisms to protect against violations and the provision of avenues for redress if required [43].

To comprehensively address structural barriers to HIV requires attention to a multitude of elements but the salience of the legal and policy environment for the lives of people who use drugs, men who have sex with men and sex workers is without question. This analysis was, however, limited by the data available in the NCPI, which covers some aspects of laws, policies and human rights but is by no means comprehensive. It also does not shed light on the degree to which reported laws are actually implemented and does not assure that what is reported is actually in place.

Laws and policies about which the NCPI does not collect information, but which are collected by other reliable sources, such as HIV-related travel restrictions and the criminalization of HIV transmission also pose enormous barriers for these and other key populations [44,45]. Recognition of the harms inherent in all of these laws, and the potential positive role that a supportive legal environment can play, suggests the need for legal reform to ensure an enabling legal and policy environment within which HIV services can be delivered more effectively and efficiently used by the populations who need them.

Several methodological issues are suggested for further research. In particular, work is needed to make the associations clearer between data on the legal and policy environment and HIV outcomes. Teasing apart how the legal and policy environment might constitute structural barriers or interventions is particularly complicated where there are conflicts between the laws and policies in place. There is a long and complex causal pathway to determining HIV outcomes; attributing them solely to the presence or absence of a specific law or policy would be simplistic. Yet, there is no denying that law plays a significant role in the ability to access HIV-related information and services.

In addition to theoretical advances, efforts are being made to develop analytical methods to assess the possible connections between legal and policy environments and HIV-related outcomes, with the aim not to determine statistical associations but to highlight general trends [46]. This work raises a host of issues for consideration including how to assess the content of law, as well as, importantly, its implementation. Although classical experimental study designs might be appropriate for evaluating some interventions to address structural barriers, practical and ethical reasons have precluded their use in instances such as legal reform [18]. Grounded within the human rights imperative for ensuring a supportive legal and policy environment, innovations in analytical methodology are required to better understand the mechanisms through which legal and policy interventions operate to affect HIV-related outcomes. Qualitative studies can also play an important role in addressing research gaps in this area.

## Conclusions

The NCPI constitutes the largest publicly available dataset on laws and policies relevant to HIV.^4^ As such it can be used to help understand the impact of structural barriers and protective laws on HIV responses, as well as to target structural interventions tailored to country level needs.

These data provide important insight into how countries understand and explain how their national laws and policies might constitute structural barriers and/or interventions affecting the effectiveness of HIV-related services with specific attention to disproportionately affected populations. Increasing recognition of these barriers by governments and civil society is a positive step. Barriers persist to collecting data on key populations which impede true understanding of the availability, accessibility, acceptability and quality of services and raise doubts regarding the validity of existing data [5,47]. Further efforts are needed to improve the quality and availability of such data to support governmental efforts to ensure that their legal and policy environment supports effective national HIV responses across all populations, and to enable civil society to mount advocacy campaigns targeting such action. A useful target for government and civil society action going forward may be National HIV Strategic Plans which could be used to push for law reform based on reported data.

Moving beyond laws and policies, further efforts are required to determine how to effectively capture information on the range of structural barriers which have been identified in the literature. Understanding the impact of different barriers, as well as the structural interventions put in place to address them, will remain a complicated task. While the NCPI provides invaluable information on national legal and policy environments, concurrent consideration of a range of indicators designed to capture the impact of structural interventions more broadly would seem a particularly appropriate next step. This would constitute a useful tool for moving forward this work and ultimately for ensuring that the human rights of key populations in national HIV responses are respected, protected and fulfilled.

## Footnotes

1 The NCPI does not specify whether these data are to include female, male and/or transgender sex workers. Most countries reported data relating solely to female sex workers, others did not specify and still others reported also on male and transgender sex workers.

2 As stated in the NCPI non-governmental sources include civil society organizations, bilateral agencies and United Nations organizations.

3 For further information on the NCPI reporting process see: *Monitoring the declaration of commitment on HIV AIDS: Guidelines on construction of core indicators, 2010 reporting*. Geneva: Joint United Nations Programme on HIV/AIDS (UNAIDS) and United Nations General Assembly Special Session on HIV/AIDS (UNGASS). Retrieved from: http://data.unaids.org/pub/manual/2009/jc1676_core_indicators_2009_en.pdf.

4 In 2012, the name of this tool was changed from National Composite and Policy Index to the National Commitments and Policy Instrument.
